# Disability, Physical Inactivity, and Impaired Health-Related Quality of Life Are Not Different in Metabolically Healthy vs. Unhealthy Obese Subjects

**DOI:** 10.3390/nu8120759

**Published:** 2016-11-25

**Authors:** Lorenzo M. Donini, Gianluca Merola, Eleonora Poggiogalle, Carla Lubrano, Lucio Gnessi, Stefania Mariani, Silvia Migliaccio, Andrea Lenzi

**Affiliations:** 1 Department Experimental Medicine-Medical Physiopathology, Food Science and Endocrinology Section, Sapienza University of Rome, Rome 00185, Italy; dottgianlucamerola@gmail.com (G.M.); Eleonora.poggiogalle@uniroma1.it (E.P.); carla.lubrano@uniroma1.it (C.L.); lucio.gnessi@uniroma1.it (L.G.); Stefania.mariani@uniroma1.it (S.M.); andrea.lenzi@uniroma1.it (A.L.); 2 Department of Movement, Human and Health Sciences, Foro Italico University of Rome, Rome 00197, Italy; silvia.migliaccio@uniroma4.it

**Keywords:** metabolically healthy obesity, disability, physical activity, quality of life

## Abstract

Background: Obesity represents a major health hazard, affecting morbidity, psychological status, physical functionality, quality of life, and mortality. The aim of the present study was to explore the differences between metabolically healthy (MHO) and metabolically unhealthy (MUO) obese subjects with regard to physical activity, disability, and health-related quality of life (HR-QoL). Methods: All subjects underwent a multidimensional evaluation, encompassing the assessment of body composition, metabolic biomarkers and inflammation, physical activity level (IPAQ questionnaire), disability (TSD-OC test), and HR-QoL (SF-36 questionnaire). MHO and MUO were defined based on the absence or the presence of the metabolic syndrome, respectively. Results: 253 subjects were included (54 men and 199 women; age: 51.7 ± 12.8 vs. 50.3 ± 11.7 years, *p* = 0.46; BMI: 38.1 ± 5.7 vs. 38.9 ± 6.7 kg/m^2^, *p* = 0.37). No significant difference was observed in body composition. There was no difference between MHO and MUO considering inflammation (hs-CRP: 6517.1 ± 11,409.9 vs. 5294.1 ± 5612.2 g/L; *p* = 0.37), physical inactivity (IPAQ score below 3000 METs-min/week in 77.6% of MHO vs. 80% of MUO subjects; *p* = 0.36), obesity-related disability (TSD-OC score > 33%, indicating a high level of obesity-related disability, in 20.2% of MHO vs. 26.5% of MUO subjects; *p* = 0.28), and the HR-QoL (SF-36 total score: 60 ± 20.8 vs. 62.8 ± 18.2, *p* = 0.27). Discussion and Conclusion: The metabolic comorbidity and the impairment of functional ability and psycho-social functioning may have a different timing in the natural history of obesity. Alterations in the physical activity level and mobility disabilities may precede the onset of metabolic abnormalities. (Trial registration 2369 prot 166/12—registered 23 February 2012; Amendment 223/14—registered 13 February 2014).

## 1. Introduction

Obesity represents one of the major health hazards since it severely affects morbidity, psychological status and physical functionality, quality of life, and mortality. Around 3 million obese people die each year worldwide, and more than 2% of global Disability-Adjusted Life Year (DALY), a measure of overall disease burden, expressed as the number of years lost due to ill-health, disability, or early death, are caused by overweight or obesity (http://www.who.int/gho/ncd/risk_factors/obesity_text/en/) [1].

Current data show that the worldwide prevalence of obesity has nearly doubled between 1980 and 2014. In 2014, more than 1.9 billion adults (18 years and older) were overweight. Of these over 600 million were obese while 42 million children under the age of 5 were overweight or obese in 2013 (http://www.who.int/mediacentre/factsheets/fs311/en/).

However, a significant portion (approximately up to 40%) of obese subjects seems to be more resistant to cardiovascular and metabolic consequences related to excess fat. Despite increased adiposity, the so-called “metabolically healthy obese” (MHO) subjects are characterized by a favorable metabolic profile (high levels of insulin sensitivity, a low prevalence of hypertension, and favorable lipid and inflammation profiles) and show similar risk of all-cause mortality and mortality for cardiovascular diseases (CVD) when compared to metabolically healthy normal-weight individuals. In a large prospective study, the risks of all-cause and CVD mortality were 57% and 76% lower in MHO compared with metabolically unhealthy obese (MUO) subjects, respectively [2].

However, the metabolic phenotype in MHO subjects is not homogeneous due to the use of discrepant definitions, and the long-term health consequences of metabolically healthy obesity remain unclear [3,4,5,6,7].

Furthermore, the clinical phenotype of obese subjects is not limited to metabolic and cardiorespiratory aspects. Physical functionality and health-related quality of life also contribute to the definition of the health status in obese subjects [8].

The aim of the present study was, therefore, to explore the differences between MHO and MUO subjects with regard to physical activity, disability, and health-related quality of life (HR-QoL).

## 2. Methods

Study participants were recruited among subjects referring to the CASCO High Specialization Center for the Care of Obesity at the Department of Experimental Medicine, Sapienza University of Rome, Italy, from September 2014 to December 2015.

Inclusion criteria were as follows: age: >18 and <65 years; body mass index (BMI): ≥30 kg/m^2^; ethnicity: Caucasian Italian.

Exclusion criteria were as follows: any malignant disease during the last 5 years; any inflammatory or autoimmune disease; corticosteroids for systemic use; any medication potentially affecting body weight or body composition; syndromic obesity; participation in a weight loss program in the last 3 months; renal failure; heart failure; history of viral or autoimmune liver disease or any other chronic liver disease; excessive alcohol intake (≥140 g/week for men or 70 g/week for women).

The study protocol was approved by the Ethics Committee of the Sapienza University of Rome, and written informed consent was obtained from all participants.

All subjects underwent a multidimensional evaluation, which was performed as follows.

Anthropometric measurements: Body weight, height, and waist circumference were measured following the procedures described in the “Anthropometric Standardization Reference Manual”. An inter-assessor alignment training session preceded the measurements. The same tools were used in all subjects: a SECA scale (Hamburg, Germany) (200 kg; to the nearest 0.1 kg, certified and homologated as class III), a flexible metallic tape (200 cm; to the nearest 0.1 cm), and a telescopic stadiometer (200 cm; to the nearest 0.1 cm). Body mass index was calculated as body weight (kg) divided by height squared (m^2^). Obesity was defined as a BMI ≥ 30 kg/m^2^ [9].

Body composition: Fat mass (FM) and lean body mass (LBM) were assessed by dual-energy-X-ray absorptiometry (DXA) (Hologic 4500 RDR, Hologic Inc., Marlborough, MA, USA), with a coefficient of variation of <1.5% for FM and LBM.

Clinical biochemistry: Blood samples were collected after an overnight fast. The following biochemical parameters were assayed: total cholesterol, HDL-cholesterol, LDL-cholesterol, triglyceride, fasting glucose and insulin, serum highly sensitive C-reactive protein (hs-CRP) levels, using commercial kits.

Inflammation: hs-CRP concentrations >3000 g/L have been shown to correspond to a high relative risk of future cardiovascular events [10].

Glucose metabolism and insulin resistance/sensitivity: All participants underwent an oral glucose tolerance test (OGTT) considering both glucose and insulin response. Homeostasis model assessment of insulin resistance (HOMA-IR) was calculated from fasting plasma insulin and glucose levels using the formula: insulin × glucose/22.5 (mU/L × mmol/L). Insulin sensitivity was assessed as the insulin sensitivity index (ISI) calculated using the OGTT values as proposed by Matsuda and DeFronzo [11]. Threshold values to indicate insulin resistance were HOMA-IR > 2.5 and Matsuda Index < 3 [12,13,14].

Metabolic syndrome: The definition proposed by the National Cholesterol Education Program—Third Adult Treatment Panel (NCEP-ATP III) was used, requiring at least three or more of the following criteria: triglycerides ≥150 mg/dL or use of lipid-lowering drugs, systolic blood pressure (BP) ≥130 mmHg, diastolic BP ≥85 mmHg or use of antihypertensive agents, glucose ≥100 mg/dL or use of medications for diabetes, HDL-cholesterol <40 mg/dL in men or <50 mg/dL in women, and waist circumference >102 cm in men or >88 cm in women [15].

Metabolically healthy or unhealthy obesity (MHO or MUO, respectively) was defined as the absence or the presence of the metabolic syndrome, respectively, as defined above.

Physical activity: The level of physical activity (PAL) during leisure time was assessed by the International Physical Activity Questionnaire (IPAQ). Based on the guidelines for data processing and analysis of the IPAQ, total scores for physical activity extracted in metabolic equivalents (METs) per minutes per week, were computed; the IPAQ score below 3000 METs-min/week indicates a low-moderate PAL [16].

Disability: A short-form questionnaire for obesity-related disabilities (TSD-OC test) proposed by the Italian Society of Obesity was filled out by all study participants. It is composed of 7 sections with a total of 36 items (pain: 5 items; stiffness: 2 items; activities of daily living and indoor mobility: 7 items; housework: 7 items; outdoor activities: 5 items; occupational activities: 4 items; social life: 6 items). Patients were asked to subjectively assess their difficulty in each item using a 0–10 visual analogue scale (10 indicating the highest level of disability and 0 indicating no difficulties in performing the task). The total score (0 to 360) represents the severity of the disability status. A TSD-OC score, higher than 33%, is consistent with a high level of obesity-related disability [17].

Health-related quality of life (HR-QoL) The Short-Form-36 (SF-36) questionnaire was administered. The SF-36 measure is a 36-item self-completed questionnaire exploring eight dimensions of health: physical functioning, role limitation due to physical problems, role limitation due to emotional problems, social functioning, mental health, energy/vitality, bodily pain, and general health perceptions. The 36 items are grouped into two summary measures: the physical composite score (PH) and the mental composite score (MH). The scores of the scales range between 0 and 100, with higher scores reflecting greater health-related quality of life, while PH and MH are norm-based scores with a mean of 50 and a standard deviation of 10 [18].

## 3. Statistical Analysis

Data are presented as mean ± standard deviation (SD) or percentage, as appropriate. Continuous variables were evaluated for distributional patterns and assumptions, and were log-transformed when appropriate. A *T*-test was used and an ANCOVA was performed for adjustment for covariates, as specified in the text and tables. Pearson χ^2^ was used for a comparison of the distribution of categorical variables. Differences were considered to be statistically significant when *p* < 0.05. Statistical analysis was performed using SPSS statistical software (SPSS Inc., Wacker Drive, Chicago, IL, USA).

## 4. Results

Two hundred fifty-three obese subjects were included (54 men and 199 women; mean age: 51.7 ± 12.8 vs. 50.3 ± 11.7 years, *p* = 0.46; mean BMI: 38.1 ± 5.7 vs. 38.9 ± 6.7 kg/m^2^, *p* = 0.37). Men had a higher waist circumference (122.4 ± 11.5 vs. 116.1 ± 17.1 cm; *p* = 0.002) and lean body mass (71 ± 13.7 vs. 55 ± 9.9 kg; *p* < 0.001) than women. The physical activity level according to the IPAQ score was not different between genders, whereas the level of disability was higher in women than men (TSD-OC score: 25.5 ± 21 vs. 11.6 ± 12.5 respectively; *p* < 0.001). Scores at the physical health (55.5 ± 19.6 vs. 71.6 ± 16.9) and mental health (57.3 ± 18.2 vs. 69.5 ± 17.2) subscales, as well as the SF-36 total score, were significantly lower (58.2 ± 19 vs. 74.1 ± 14.6) in women than men (all *p* < 0.001). The prevalence of metabolic syndrome, and therefore of MUO, was 59.7% (79.6% in men vs. 54.3% in women, respectively; *p* = 0.001).

Study participants’ characteristics according to metabolic health are described in Table 1. MHO subjects were younger than MUO individuals (47.9 ± 11.9 vs. 52.5 ± 11.7 years; *p* = 0.001), with a lower BMI (*p* < 0.05 in both gender). No statistically significant difference was observed in terms of waist circumference and body composition parameters (body fat, fat mass, and lean body mass) in both genders, after adjustment for age and BMI.

As expected, MHO subjects, compared with their MUO counterparts, exhibited a lower prevalence of hypertension (38.6% vs. 88%; *p* < 0.001), elevated glycaemia (7.9% vs. 72.5%; *p* < 0.001), insulin resistance according to HOMA-IR (38.8% vs. 67%; *p* < 0.001), and Matsuda Index (7.5% vs. 21.2%; *p* = 0.046), hypertriglyceridemia (3% vs. 44.9%; *p* < 0.001), and reduced HDL levels (16.7% vs. 61.5%; *p* < 0.001). There was no difference for inflammation described by hs-CRP levels (6517.1 ± 11,409.9 vs. 5294.1 ± 5612.2 g/L; *p* = 0.37), and more than 50% of subjects in both groups were above the threshold of 3000 g/L for hs-CRP (*p* = 0.83). 

**Figure 1:**
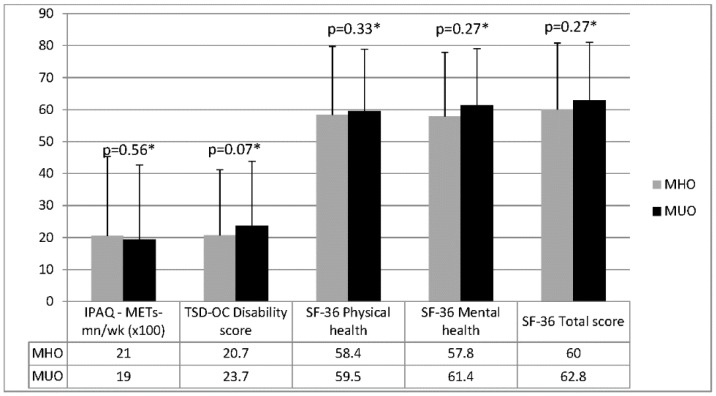
Physical activity level, disability, and health-related quality of life parameters in metabolically healthy obese (MHO) and metabolically unhealthy obese (MUO) subjects. Legend: *IPAQ:* International Physical Activity Questionnaire; TSD-OC: Test SIO for obesity-related disability: SF-36: Short-Form-36 questionnaire for health-related quality of life; * after adjustment for age, gender, and BMI.

**Figure 2:**
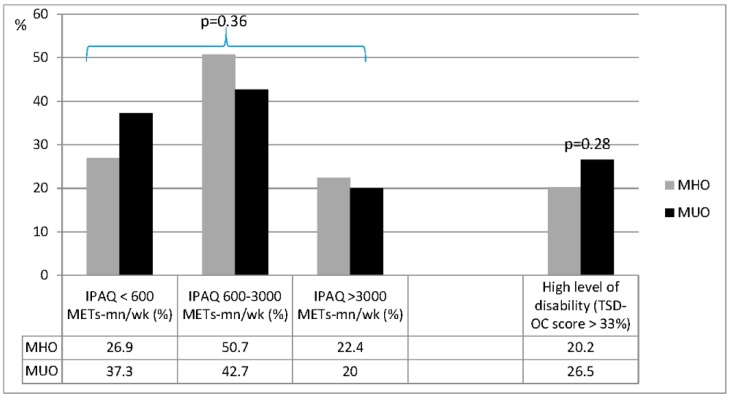
Prevalence of disability and physical inactivity in metabolically healthy obese (MHO) and metabolically unhealthy obese (MUO) subjects. IPAQ: International Physical Activity Questionnaire; TSD-OC: Test SIO for obesity-related disability.

The levels of physical activity, obesity-related disability, and the health-related quality of life were not significantly different between MHO and MUO subjects (Figure 1). A TSD-OC score higher than 33%, indicating a high level of obesity-related disability, was present in 20.2% of MHO individuals and 26.5% of MUO subjects (*p* = 0.28); the IPAQ score was below 3000 METs-min/week in 77.6% of MHO participants and in 80% of MUO subjects (*p* = 0.36). In addition, the perceived health-related quality of life was not different between groups (SF-36 total score: 60 ± 20.8 vs. 62.8 ± 18.2; *p* = 0.27), with no difference in the scores obtained at the mental and physical health subscales (Figure 2).

## 5. Discussion

In the present study, MHO and MUO subjects were characterized by a similar phenotype in terms of physical activity and disability levels, with analogous impairment in quality of life and similar body composition.

Although a universally accepted definition of the MHO phenotype has not been established, the significant reduction of risk for cardiovascular diseases (CVD) and type 2 diabetes has been partly attributed to a favorable metabolic profile, despite excess body fat. However, the pathophysiological factors related to the protective metabolic characteristics in MHO individuals are not thoroughly understood [19,20,21].

In a recent review of the literature, 30 definitions were identified in 27 different studies to describe metabolic health [22]. The most frequently used definition of MHO was based on at least four components of the metabolic syndrome (blood pressure, HDL-cholesterol, fasting plasma glucose, and triglyceride levels), with cut-off values from the NCEP-ATPIII definition [22]. Moreover, the use of other parameters like inflammatory status or insulin resistance does not seem to improve the efficacy of the definition of MHO to assess cardio-metabolic risk [7,23].

Therefore, in the present study, we defined metabolic health relying on the classification adopted by the NCEP-ATP-III expert panel since these criteria and their respective threshold values for risk assessment have been validated in multiple occasions. In fact, whether inflammation or insulin resistance account for the metabolic differences observed between metabolically healthy and unhealthy individuals is relatively unknown. In our study cohort, hs-CRP levels were not significantly different in MHO vs. MUO subjects, and more than 50% of both groups showed a level of hs-CRP >3000 g/L, indicating a high level of inflammation.

In our study, the prevalence of MHO subjects was 40.3%. In a recent paper, Hinnohuo et al. showed that the prevalence of MHO subjects ranged from 9% to 41% [7], with prevalence estimates varying widely due to the definitions used and to the lack of consensus to describe metabolic health [24,25,26].

The major question regarding metabolically healthy obesity is whether MHO subjects will evolve through the appearance of risk factors and, then, the development of cardiometabolic diseases. Different longitudinal studies addressed this research question: two of them demonstrated an increased incidence of diabetes in MHO subjects after a follow-up of 11 and 20 years [27,28], while in another study (9-year follow-up), an increased risk for all-cause mortality was found in obese subjects regardless of their metabolic status [12]. According to four out of the five definitions used in their study, Hinnohuo and coworkers showed that both MHO and MUO participants had an elevated mortality risk compared with metabolically healthy normal-weight individuals [7]. Bobbioni–Harsch et al. reported that 57.2% of subjects who were MHO at baseline developed one or more cardiometabolic risk factors after three years [29]. In a systematic review and meta-analysis including eight studies (*n* = 61,386; 3988 events), Kramer et al. reported that MHO subjects had an increased risk for cardiovascular events compared with metabolically healthy normal-weight individuals when considering studies with 10 or more years of follow-up (RR = 1.24; 95% CI, 1.02 to 1.55) [30]. Finally, even in the absence of metabolic abnormalities and compared with metabolically healthy normal-weight individuals, obese subjects are at increased risk for adverse long-term outcomes, supporting the concept that there is no healthy pattern of augmented weight and adiposity [12,29,30].

Direct comparison of our observations with findings from other studies is made difficult by the use of different study populations (combining normal weight and overweight subjects in most cases) and different definitions of health risks and/or metabolic health. Few studies have considered functional aspects in obese subjects. Indeed, obesity is a clinical condition characterized by significant clinical implications, such as co-morbidities and somatic fragility, which seriously affect functional independence, psychological wellbeing, and overall quality of life at all ages.

The majority of the studies analyzed the influence of obesity on mortality, while other aspects of health need to be considered [31]. Diehr et al. suggested that the desirable weight should be defined on the basis of alternative outcomes, such as disability or risk of disease [32]. Few studies have taken into account the effects of obesity on health outcomes such as disability: the association was at least comparable to the association between obesity and mortality. In fact, years lost to disability (represented by incident and persistent functional impairment) increase among obese patients compared with normal weight participants and overcome the mortality effects [33,34,35]. 

Excluding underweight subjects, the prevalence of disability tends to increase in obese individuals as their BMI increases. In addition, the risk of mobility limitations, physical functionality impairment and of limitations in the activities of daily living (ADL) is substantially higher among obese subjects compared with those who are normal weight. The analysis of pooled data from adult subjects participating in the 2003–2009 National Health Interview Survey (NHIS) showed that disability was present in 41% of obese subjects and in 27% of normal-weight adults. Disability was related to mobility difficulties in all classes of BMI but in obese adults it was also related to a higher prevalence of social and work limitations [36]. In our sample, the prevalence of disability was 20.2% in MHO and 26.5% in MUO subjects.

Finally, disabled people face a higher mortality risk than nondisabled people do regardless of the cause of disability [37,38]. The difference can be explained by diseases and other risk factors for those with mild disability, but it is plausible that more severe disabilities have an independent effect on mortality [39].

In our study, we used the TSD-OC tool to evaluate the disability level. This is the only instrument validated for this purpose in obese subjects, and it represents a reliable and valid tool for measuring self-reported disability in obesity. In the validation study, the TSD-OC measure showed an excellent stability at the test–retest (*r* = 0.90) and an excellent internal consistency (Cronbach’s > 0.90). Moreover, a significant correlation was found with SF-36 scores, the distance walked at the 6 min walk test, and grip strength, and a total of 26 ICF (International Classification of Functioning, Disability and Health) categories, mostly related to the area of mobility, were correlated to TSD-OC score [17].

Physical activity is associated with disability independent of obesity, which in turn is responsible for sedentariness and, as we reported in the present study, disability [39]. Many studies showed that sedentary behavior represents an independent risk factor for several adverse health outcomes (i.e., cancer, cardiovascular diseases, and diabetes) and mortality (premature, CVD-related, and all-cause mortality) [40,41]. In our study population, almost 80% of MHO subjects, a percentage similar to MUO patients, had a low-moderate level of physical activity (below 3000 MET-mn/week) according to the IPAQ score, and this prevents considering them healthy or at low risk of morbidity/mortality in spite of their metabolic profile.

The link between obesity and disability may be explained through multiple pathways. Obesity is associated with different clinical conditions that may affect functional autonomy: osteoarthritis of the weight-bearing joints, diabetes mellitus, and respiratory and cardiovascular diseases. In addition, muscle strength per unit of skeletal muscle mass declines with increasing adiposity, even though lean body mass increases in parallel with fat mass in obese individuals [31]. Furthermore, biomechanical implications of excess weight also need to be taken into account: alterations in movement coordination may occur in people with high BMI as an attempt to maintain stability, but these movement adaptations may be responsible for walking difficulties and may lower the level of safety in physical activity and physical performance [42,43,44].

In our study population, though we observed a different BMI in MHO compared with MUO subjects, body composition (body fat, fat mass, and lean body mass) was not different after adjusting for BMI, age, and gender.

A wealth of studies have demonstrated that obesity negatively affects quality of life. The degree of obesity, the level of disability, psychological status, especially the presence of depression and anxiety, and the level of comorbidity, may contribute to the low perceived QoL. In our study population, low scores at different sections (physical and mental health) and at the global scores obtained at the SF-36 test were reported in both MHO and MUO subjects. Therefore, the absence of metabolic abnormalities did not positively affect the perceived level of QoL. The World Health Organization indicated that the health status cannot be measured only considering morbidity or mortality [45], but it is a multidimensional construct including physical, mental, and social domains. Therefore, low levels of perceived QoL are consistent with deteriorated health status [46,47].

All participants in the present study were recruited in a clinical setting devoted to the treatment of obesity, among patients seeking care for obesity. Our results need to be verified in larger cohorts as well as in the general population. In addition, we used the IPAQ score for assessing the physical activity level. Even validated, the IPAQ, as other self-reported measures, can be affected by misreporting.

## 6. Conclusions

To conclude, our findings suggest that obesity-related alterations in different domains, mainly the metabolic comorbidity versus the functional ability and psycho-social functioning, may have a different timing in the natural history of obesity; in particular, alterations in the physical activity level and mobility disabilities may precede the onset of metabolic abnormalities. The use of the term “metabolically healthy obesity” may lead clinicians to underestimate obesity-related sequelae other than metabolic and cardiovascular diseases, and the impact of obesity on the overall health status. A multidimensional approach, considering the multifaceted clinical features of excess adiposity, could provide beneficial effects in terms of obesity care and obesity-related health risk management [48,49].

## Figures and Tables

**Table 1:** Clinical and functional characteristics related to metabolic health of obese subjects.

				MHO (*n* = 102)	MUO (*n* = 151)	*p*
	Age (years)			47.9 ± 11.9	52.5 ± 11.7	0.001
Anthropometry and body composition	BMI	Males	kg/m^2^	34.5 ± 4.4	39 ± 5.7	0.02
		Females	kg/m^2^	37.7 ± 6.2	39.8 ± 7	0.03
		BMI 30–34.9 kg/m^2^ (%)		46.1	23.2	<0.001
		BMI 35–39.9 kg/m^2^ (%)		53.9	76.8	
	Waist circumference	Males	cm	114.2 ± 10.9	124.5 ± 10.8	0.14 **
		Females	cm	113.5 ± 19.1	118.1 ± 15.1	0.69 **
		WC >102 cm in Males or >88 cm in Females (%)		97	99.3	0.14
	Body fat	Males	%	27.1 ± 5.1	30.4 ± 4.1	0.56 **
		Females	%	39.9 ± 3.8	39 ± 4.1	0.02 **
	Fat mass	Males	kg	27.6 ± 8.2	34 ± 7	0.4 **
		Females	kg	38.8 ± 9	38.3 ± 10	0.007 **
	Lean body mass	Males	kg	70.4 ± 6.3	71.1 ± 14.7	0.76 **
		Females	kg	55.3 ± 9	54.7 ± 10.6	0.12 **
Clinical status	Blood pressure	SBP (mmHg)		124.3 ± 12.4	132.7 ± 12.8	0.001 *
		DBP (mmHg)		78.9 ± 9.8	83.3 ± 10	0.02 *
		Hypertension (≥130/85 mmHg) ^§^ (%)		38.6	88	<0.001
	Glycaemia	mg/dL		91.2 ± 15.4	105.6 ± 25.2	<0.001 *
		Glycaemia ≥ 100 mg/dL ^§§^ (%)		7.9	72.5	<0.001
	HOMA-IR	Score		2.5 ± 2.1	5.8 ± 9.2	<0.001 *
		HOMA-IR > 2.5 (%)		38.8	67	<0.001
	Matsuda ISI	Index		7.3 ± 5	5.4 ± 3.4	0.08 *
		Matsuda index < 3 (%)		7.5	21.2	0.046
	Triglycerides	mg/dL		99.1 ± 13.5	161.8 ± 93.5	<0.001 *
		Triglycerides 1 ≥ 50 mg/dL ^§§§^ (%)		3	44.9	<0.001
	HDL	mg/dL		55.6 ± 11.8	46.6 ± 11.3	<0.001 *
		HDL < 40 mg/dL (M) or <50 mg/dL (F) ^§§§^ (%)		16.7	61.5	<0.001
	hs-CRP	μg/L		6517.1 ± 11,409.9	5294.1 ± 5612.2	0.37 *
		hs-CRP > 3000 μg/L		55.7	54	0.83
Physical functioning and quality of life	IPAQ	METs-mn/week		2055.3 ± 2474.7	1932.8 ± 2334.4	0.56 *
		IPAQ < 600 METs-mn/week (%)		26.9	37.3	0.36
		IPAQ 600–3000 METs-mn/week (%)		50.7	42.7	
		IPAQ > 3000 METs-mn/week (%)		22,4	20	
	TSD-OC	Disability score		20.7 ± 20.5	23.7 ± 20.1	0.07 *
		>33% (%)		20.2	26.5	0.28
	SF-36	Physical health		58.4 ± 21.4	59.5 ± 19.4	0.33 *
		Mental health		57.8 ± 20.1	61.4 ± 17.7	0.27 *
		Total score		60 ± 20.8	62.8 ± 18.2	0.27 *

*Legend:* MHO: metabolically healthy obese subjects; MUO: metabolically un-healthy obese subjects; BMI: body mass index; WC: waist circumference; SBP: systolic blood pressure; DBP: diastolic blood pressure; IPAQ: International Physical activity Questionnaire; TSD-OC: SIO test for obesity-related disability; hs-CRP: high sensitivity C-reactive protein; ^§^ or use of antihypertensive agents; ^§§^ or use of antidiabetics; ^§§§^ or use of antidyslipidemic agents; * after adjustment for age, gender and BMI; ** after adjustment for age and BMI.
